# Aberrant Glycosylation in Pancreatic Ductal Adenocarcinoma 3D Organoids Is Mediated by KRAS Mutations

**DOI:** 10.1155/2024/1529449

**Published:** 2024-03-18

**Authors:** Hiromitsu Nakahashi, Tatsuya Oda, Osamu Shimomura, Yoshimasa Akashi, Kazuhiro Takahashi, Yoshihiro Miyazaki, Tomoaki Furuta, Yukihito Kuroda, Pakavarin Louphrasitthiphol, Bryan J. Mathis, Hiroaki Tateno

**Affiliations:** ^1^Department of Gastrointestinal and Hepato-Biliary-Pancreatic Surgery, Faculty of Medicine, University of Tsukuba, Tsukuba 305-8575, Japan; ^2^International Medical Center, University of Tsukuba Hospital, Tsukuba, Japan; ^3^Biotechnology Research Institute for Drug Discovery, National Institute of Advanced Industrial Science and Technology, Tsukuba 305-8568, Japan

## Abstract

Aberrant glycosylation in tumor cells is a hallmark during carcinogenesis. KRAS gene mutations are the most well-known oncogenic abnormalities but their association with glycan alterations in pancreatic ductal adenocarcinoma (PDAC) is largely unknown. We employed patient-derived 3D organoids to culture pure live PDAC cells, excluding contamination by fibroblasts and immune cells, to gasp the comprehensive cancer cell surface glycan expression profile using lectin microarray and transcriptomic analyses. Surgical specimens from 24 PDAC patients were digested and embedded into a 3D culture system. Surface-bound glycans of 3D organoids were analyzed by high-density, 96-lectin microarrays. KRAS mutation status and expression of various glycosyltransferases were analyzed by RNA-seq. We successfully established 16 3D organoids: 14 PDAC, 1 intraductal papillary mucinous neoplasm (IPMN), and 1 normal pancreatic duct. KRAS was mutated in 13 (7 G12V, 5 G12D, 1 Q61L) and wild in 3 organoids (1 normal duct, 1 IPMN, 1 PDAC). Lectin reactivity of AAL (*Aleuria aurantia*) and AOL (*Aspergillus oryzae*) with binding activity to *α*1-3 fucose was higher in organoids with KRAS mutants than those with KRAS wild-type. *FUT6* (*α*1-3fucosyltransferase 6) and *FUT3* (*α*1-3/4 fucosyltransferase 3) expression was also higher in KRAS mutants than wild-type. Meanwhile, mannose-binding lectin (rRSL [*Ralstonia solanacearum*] and rBC2LA [*Burkholderia cenocepacia*]) signals were higher while those of galactose-binding lectins (rGal3C and rCGL2) were lower in the KRAS mutants. We demonstrated here that PDAC 3D-cultured organoids with KRAS mutations were dominantly covered in increased fucosylated glycans, pointing towards novel treatment targets and/or tumor markers.

## 1. Introduction

To discover new frontiers in the fight against relentless pancreatic ductal adenocarcinoma (PDAC), it is necessary to explore targets beyond DNA, RNA, and proteins, which have been well studied to date [1]. For this reason, glycans, the third life chain after nucleic acids and proteins, are an important post-translational modification and present new opportunities for targeting [2]. Moreover, as glycans constitute the outermost layer of cells, diverse alterations during carcinogenesis affect the multiple processes of invasion and metastasis, making glycosylation a keystone mechanism in cancer progression [1, 3]. While several glycosylation changes have been reported in PDAC [4], the mechanism of glycosylation change in PDAC remains largely unknown.

In the multistep genesis and progression of PDAC, four major driver genes (KRAS, TP53, SMAD4, and CDKN2A) have been well highlighted [5]. Among these, the KRAS mutation is the most frequent genetic mutation and is present in approximately 90% of PDAC cases while also occurring in the earliest stages of low-grade PanIN IA lesions [6–8]. KRAS activation has been reported to function as a master regulator, affecting the properties of multiple tumor microenvironment components and promoting cancer progression by mobilizing cells such as fibroblasts, macrophages, neutrophils, and lymphocytes [7, 9, 10]. We considered that profiling of the aberrant glycosylation associated with KRAS mutations could be useful for elucidating the mechanisms of PDAC progression as well as for developing early diagnostic markers and novel anticancer therapeutic targets. However, any associations between KRAS mutations and glycans in PDAC remain unknown.

A signature challenge in glycan analysis of clinical samples is the difficulty of eliminating the contaminating influence of noncancer components such as stroma and immune cells [11–13]. Obtaining isolated epithelial samples of PDAC for glycan analysis, without high ratios of these noncancer contaminants, is particularly difficult. Furthermore, simultaneous analysis of glycans, genes, and transcriptomes is limited due to the small number of cells available from clinical specimens. To solve these problems, we focused on three-dimensional (3D) organoid technology to generate PDAC samples that preserve only epithelial cancerous cells [14]. These 3D organoids allow for specific epithelial amplification by serving as a biologically relevant structure with polarized cells that recapitulate tumor morphology and biology [15, 16].

In this study, we established patient-derived organoids from PDAC surgical specimens and analyzed the glycan status in these organoid-derived tumor cells. We analyzed the relationship between altered glycans and glycan-related genes, with a particular focus on the status of KRAS mutations.

## 2. Materials and Methods

### 2.1. Patient Information

**Figure 1:**
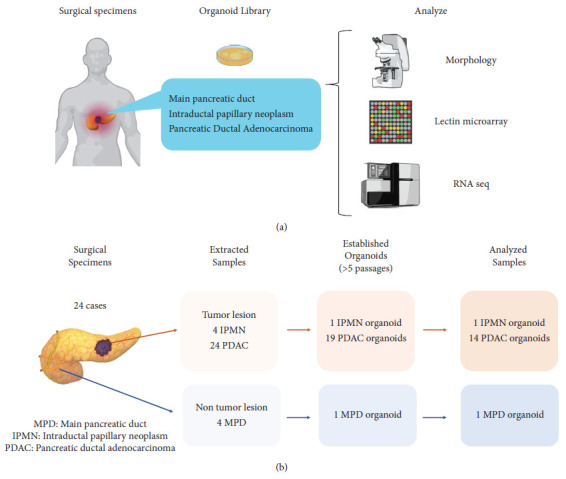
(a) Experimental scheme. Diagram of steps for the glycosyltransferase and glycan analysis of patient-derived organoids. (b) Flow diagram of sample collection. Samples were collected from 24 patients. Collected and cultured tissues were IPMN (*N* = 4), PDAC (*N* = 24), and normal pancreatic ducts *N* = 4. Organoids successfully established were IPMN (*N* = 1), PDAC (*N* = 19), and normal pancreatic duct (*N* = 1). The cases that could be further analyzed were IPMN (*N* = 1), PDAC (*N* = 14), and normal pancreatic duct (*N* = 1).

The establishment of organoid libraries was approved by the Institutional Review Board of the University of Tsukuba Hospital, Japan (IRV code: H28- 90) and informed consent was obtained from all PDAC patients. Surgical specimens were obtained from a total of 24 patients who underwent surgery for PDAC from April 2020 to January 2021. The obtained samples included 24 PDACs, 4 intraductal papillary mucinous neoplasms (IPMNs), and 4 nocancerous pancreatic ducts (Figure 1(b)).

### 2.2. Establishment of Organoids

Surgical specimens were minced into 1 mm^3^ fragments after being extensively rinsed in phosphate-buffered saline (PBS). Undigested pellets were then treated with TrypLE Express (Thermo Fisher Scientific, cat# 12605010) at 37°C for 10 min after digestion with Liberase TH (Roche Diagnostics, cat# 05401151001) for 30 min. To inactivate digestive enzymes, collected epithelia were carefully washed with PBS containing 10% fetal bovine serum (FBS) before plating. The sedimented cells from the centrifuged ascite samples were used for organoid culture after three washes with ice-cold PBS. Culture media was applied over the Matrigel-embedded isolated PDAC cells. Basal culture medium was made according to a prior description [17]. Penicillin/streptomycin (FUJIFILM Wako Pure Chemical Corporation, cat# 168-23191) 10 mM HEPES (Thermo Fisher Scientific, cat# 15630080), 2 mM GlutaMAX (Thermo Fisher Scientific, cat# 35050061), 1×B27 (Thermo Fisher Scientific, cat# 17504044), 10 nM gastrin I (Sigma-Aldrich, #cat G9145), and 1 mM N-acetylcysteine (Sigma-Aldrich, #cat A9165) were added to Advanced Dulbecco's Modified Eagle's Medium/F12 (Thermo Fisher Scientific, cat# 12634010). The following factors were added to the basal culture medium to create a complete medium: 50% Afamin-Wnt-3A serum-free conditioned medium (MBL Life Science, cat# J2-001) [18], 500 nM A83-01 (Tocris, cat#2939), 100 ng/ml mouse recombinant EGF (Peprotech, cat#AF100-15), 50 ng/ml human recombinant FGF10 (Peprotech, cat# 100-26), and 100 ng/ml mouse recombinant noggin (Peprotech, cat# PEP-250-38). Plated organoids were maintained in an incubator with 5% CO_2_ and 20% O_2_, exchanging media every three to four days. For passaging, organoids were collected, washed, and disrupted by digestion with TrypLE Express. We cultivated the organoids in EGF-free media to enrich PDAC-derived cells. All cells were used for experiments between Passage 3 and 23. Molecular studies and cryopreservation were performed on the harvested cells according to the reports of Fujii et al. [19].

### 2.3. Membrane Protein Isolation and Quantification

Hydrophobic fractions containing membrane proteins were obtained using the Sigma-Aldrich CelLytic MEM Protein Extraction Kit according to the manufacturer's instructions. Protein concentrations were measured using a micro-BCA assay kit (cat#23235, Thermo Fisher Scientific). Proteins were then fluorescently labeled with the monoreactive dye Cy3 (cat#16968983, GE Healthcare, Boston, MA, USA).

### 2.4. Lectin Microarray

High-density lectin microarrays were produced as previously stated [20]. Lectin microarrays were incubated with 0.5 mg/mL Cy3-labeled protein lysates at 20°C overnight. Fluorescence images were captured using an evanescent field-activated fluorescence scanner (Bio-REX Scan 200, Rexxam Co., Ltd).

### 2.5. RNA Extraction

Organoid RNA was extracted using the RNeasy Plus Mini Kit (Qiagen, cat# 74134). Based on the RNA Integrity Number (RIN) value obtained from TapeStation 4150, RNA quality was assessed. In this investigation, only specimens with an RIN >8.0 were employed.

### 2.6. RNA-Seq Bioinformatics and Data Analysis

A sequencing library was prepared with a NEBNext Ultra II Directional RNA Library Prep Kit for Illumina. Library size distribution and yield were determined using Tape Station (Agilent), normalized, and pooled before loading and sequencing on NovaSeq 6000 (Illumina S2 Reagent v2 Kit (200 cycles)). Library preparation and sequencing were carried out using the University of Tsukuba Precision Medicine genomic service. Unmixed fastq results from multiple sequencing lanes were joined using a UNIX cat command before quality and adaptor trimming with trim_galore (v.0.6.5) (https://github.com/FelixKrueger/TrimGalore), retaining only reads >40 bp before mapping against UCSC hg38 using rna-star (v.2.7.8a) [21] with–quantMode. Feature counts for each sample set were first filtered for genes whose expression was <1 count-per-million prior to glmQLFTest for differential gene expression analyses using EdgeR (v.3.34.0) [22] on R x64 (v.4.1.0). The normalized gene expression matrix was used for all subsequent analyses. The mutation status of each organoid line was determined from piled-up reads of sorted and index bam files (samtools [v.1.9], https://github.com/samtools/) then visualized using igv (v.2.4.11) [23].

### 2.7. Immunohistochemistry

The organoids were solidified in iPGell (Genostaff, cat# PG20-1), fixed in 4% PFA, embedded in paraffin, and sliced to a thickness of 3 *μ*m. The sections were deparaffinized before antigen retrieval was performed by autoclaving sections immersed in 10 mmol/L sodium citrate buffer. The tissue sections were then treated with 3% hydrogen peroxidase in methanol for 15 minutes to inactivate endogenous peroxidase activity. Following blocking with normal bovine serum, the tissues were incubated with anti-CK19mAb (2 *μ*g/ml, Dako, cat#M0888) following primary antibodies overnight at 4°C. Tissue sections were visualized by 3,3′- diaminobenzidine (Nichirei Bioscience, cat# 425011) and counterstained with hematoxylin. Images were captured with an ECLIPSE Ti2 microscope (Nikon).

## 3. Results

### 3.1. Establishment of a Pancreatic Ductal Adenocarcinoma (PDAC) Organoid Library

Among a total of 24 attempted PDAC 3D organoids (see Table S1 for detailed characteristics of patients), 19 were recognized as established organoids since they could successfully passage more than 5 times. Likewise, 1 of 4 IPMN and 1 of 4 normal main pancreatic duct organoids were established. Among those 21 established organoids, sufficient amounts of protein and RNAs for analysis were extracted from 14 PDACs, 1 IPMN, and 1 normal PDAC. Pairings of a PDAC organoid with a non-PDAC counterpart were available from 2 patients; one was a PDAC-normal MPD pair and the other was PDAC-IPMN pair, eliminating the influence of individual variations in glycan expression status for those pairs (Figures 1(a) and 1(b)).

### 3.2. KRAS Mutation Profiling in Organoids

**Figure 2:**
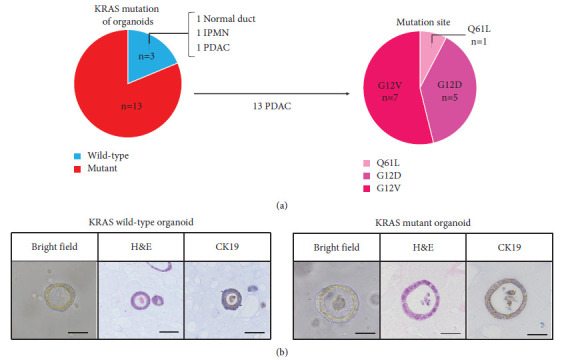
KRAS mutation profile of the organoids. (a) Red areas in the left chart indicate KRAS -positive while blue indicates KRAS wild -type. (b) Brightfield images, H&E staining and CK19 immunostaining of 2 organoids cultures are shown (left: KRAS wild -type organoid, right: KRAS mutant organoid). Scale bar: 50 *μ*m.

Among 14 PDAC organoids, KRAS mutations were found in 13 (92.9%), including 7 G12V, 5 G12D, and 1 Q61L mutations. These mutations were not found in normal pancreatic ducts, IPMN, or one PDAC case (Figure 2(a)). In morphology, there were no obvious changes between KRAS wild-type PDAC organoids and KRAS-mutant PDAC organoids, and both showed similar cystic form Figure 2(b).

To confirm that the KRAS mutations existed in our PDAC organoids, EGF selection pressure was applied. EGF is an important niche factor of the KRAS pathway, as KRAS mutants do not require EGF to survive [17]. As expected, KRAS wild-type organoids were incapable of long-term culture without EGF in the medium while all KRAS mutant organoids survived without EGF (Figure S1).

### 3.3. Lectin Microarray Comparison of Glycan Expression between KRAS Mutant and Wild-Type Organoids

**Figure 3:**
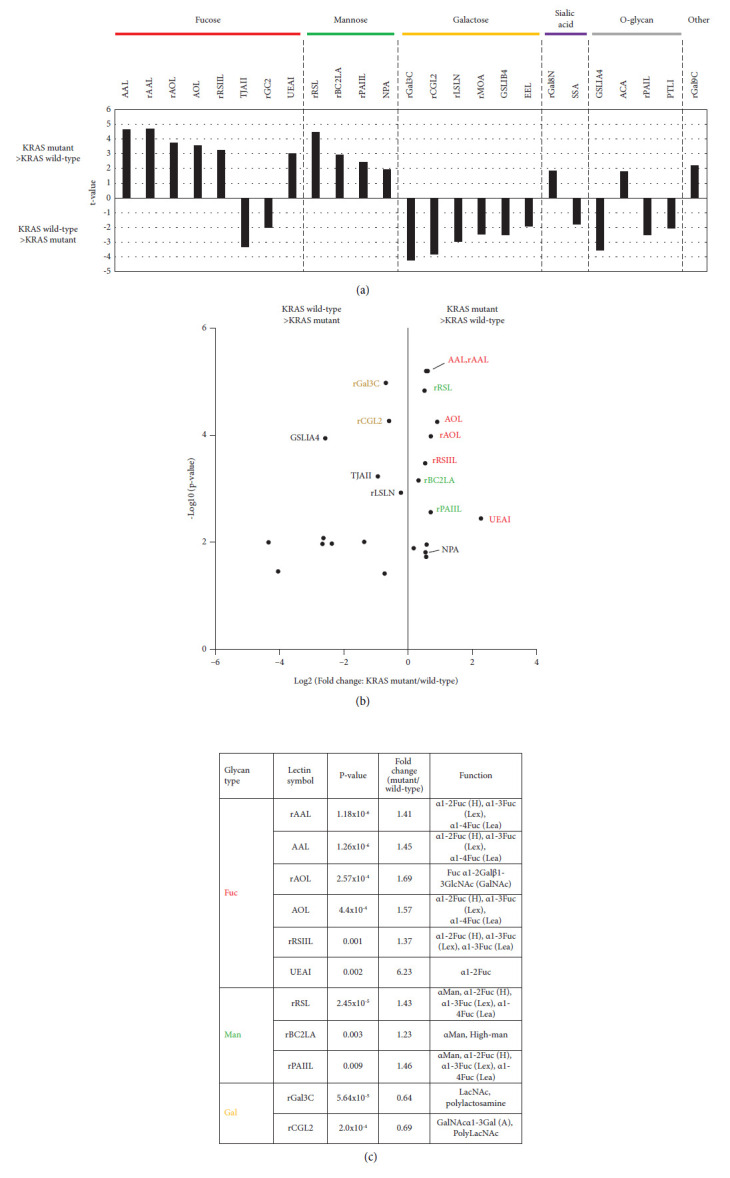
Comparison of glycan expression based on lectin microarray results between KRAS mutant and KRAS wild-type organoids. (a) List with *t* values of lectins significantly changed between KRAS mutation positive organoids and KRAS wild organoids. Labels and bars at the top of the figure indicate the type of glycan structure to which each lectin binds. Fucose (Fuc); red bar, Mannose (Man); green bar, Galactose (Gal); orange bar, sialic acid; purple bar, O-glycan; gray bar. Statistically significant differences are calculated by unpaired student's *t* test and the lectins with *p* < 0.05 are selected. Lectins are categorized based on their binding specificities. (b) The volcano plot of differential intensity of lectins in KRAS mutation organoids and KRAS wild organoids represents fold change and *p* values of the selected lectins on *x*- and *y*-axis, respectively. (Lectins listed in Figure 3(a)) (c) Lists of representative lectins that are listed by type in order of *p* value.

To identify differentially binding lectins in KRAS mutant organoids (*n* = 13) and KRAS wild-type organoids (*n* = 3), statistical analysis was performed using mean normalized signals obtained from lectin microarrays (Table S2). The results showed that several classes of lectins were significantly altered (*p* < 0.05) between KRAS mutant and wild-type organoids, and these could be classified based on their glycan binding specificity (Figure 3(a)). In the KRAS mutant organoids, 13 lectins were significantly higher compared to 12 lectins in wild-type organoids. The lectins enriched in KRAS mutants included fucose-binding lectins (AAL, rAAL, AOL, rAOL, rRSIIL, and UEAI), mannose-binding lectins (rRSL, rBC2LCA, rPAIIL, and NPA), and galactose-binding lectins (rGAL3C, rCGL2, rLSLN, rMOA, GSLIB4, and EEL). Among these highly enriched lectins, AAL, rAAL, AOL, rAOL, and rRSL were significantly elevated in the mutants (*p* < 0.001) (Figures 3(b), 3(c)).

Differences in expression between G12V, G12D, and Q61L were thus compared. Differences in lectin expression between KRAS mutation sites (G12V, G12D, and Q61L) were thus compared through signal intensities. KRAS G12V and G12D mutant organoids each showed increased expression of fucose-binding lectins (AAL, rAAL, AOL, rAOL, and rRSIIL) compared to the KRAS wild type. On the other hand, there were no significant differences between KRAS G12V and G12D in the levels of fucose-binding lectins (AAL, rAAL, AOL, and rAOL) (Figure S2(a)).

Although it is widely known that sialic acid is involved in carcinogenesis [1, 24], there were few significant changes in the sialic acid-binding lectins in this study (Figure 3(a)).

### 3.4. Comparison of Glycosylation-Related Gene Expression Based on RNA-Seq between KRAS Mutant and Wild-Type Organoids

**Figure 4:**
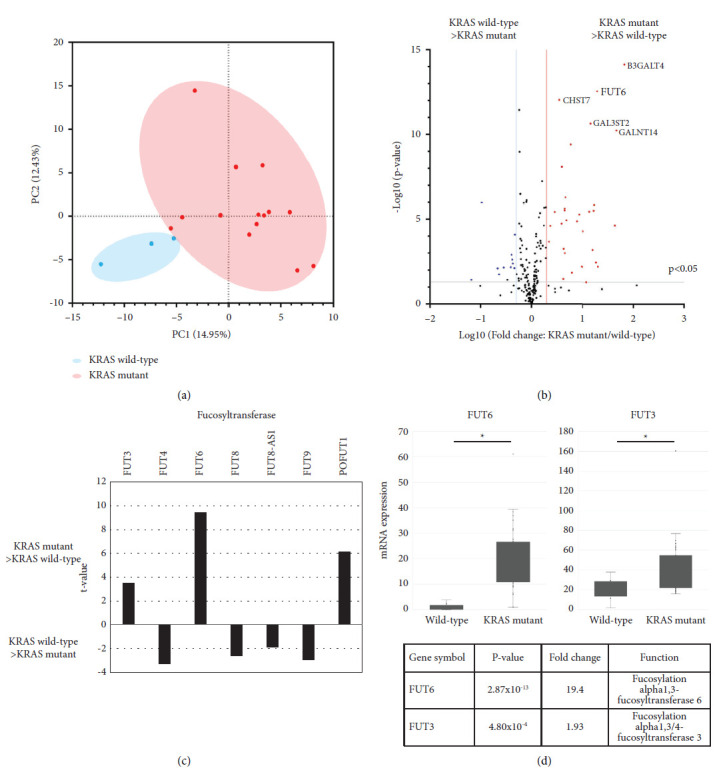
Comparison of glycosylation-related genes expression based on the result of RNA-seq between KRAS mutant and KRAS wild-type organoids. (a) The principal component analysis of gene expression data (glycosyltransferase *n* = 212) for the 16 organoids. Red and blue dots and circles are indicated to KRAS mutation positive organoids and KRAS wild organoids, respectively. (b) The volcano plot of mRNA differential expressions in KRAS mutation organoids and KRAS wild organoids represents fold change and *p* values on *x*- and *y*-axis, respectively. The vertical red and blue lines represent a fold-change cutoff of ≥2.0. (c) List with *t* values of fucosyltransferase between KRAS mutation positive organoids and KRAS wild organoids. Statistically significant differences are calculated by unpaired student's *t* test and *p* < 0.05 are selected. (d) The FUT6 mRNA expression from RNA seq, and the list of details of results. ^*∗*^*p* < 0.001.

To address which enzymes are responsible for glycan changes in KRAS mutant organoids, we performed RNA-seq and focused on glycan-related genes. Such genes, including glycosyltransferases and hydrolases, were extracted from the GlyCosmos Portal database (https://glycosmos.org/) and 212 gene expression instances were analyzed. PCA analysis showed that the KRAS wild-type organoids tended to have similar expression profiles (Figure 4(a)) while several glycan-related genes were significantly altered (*p* < 0.05) between KRAS mutant and wild-type organoids (Figure 4(b)). Among them, the fucosyltransferase genes *FUT6* and *FUT3* were significantly elevated in the KRAS mutant group (Figures 4(c), 4(d)).

We also compared the gene expression of glycosyltransferases involved in the synthesis of mannosylated, galactosylated, and sialylated glycans (Figure S3), finding that, in terms of site-specific mutation differences, *FUT6* in G12V and G12D was significantly upregulated versus the KRAS wild-type (Figure S2(b)).

### 3.5. Comparison of KRAS Mutant Wild-Type Organoids from the Same Patients

**Figure 5:**
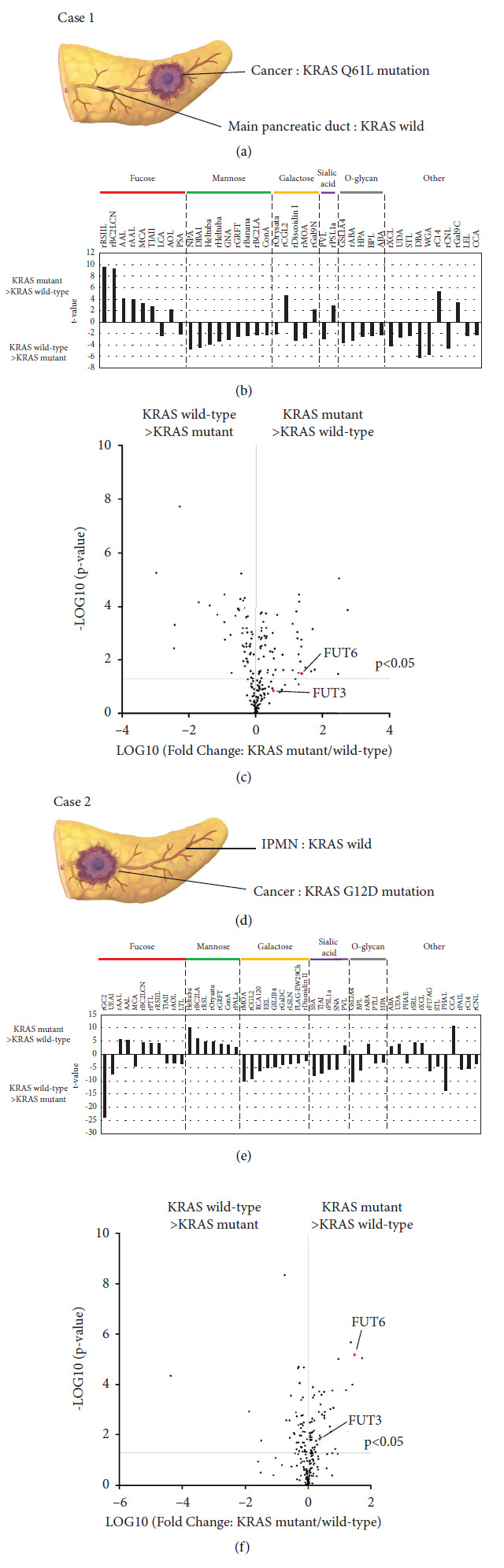
Analysis of glycosyltransferase and glycan expression in organoids established from the same patient. Case1 (a–c), Case2 (d–f). (a, d) Schema indicating the pancreatic lesion of Case1 used for the organoid and mutation site of each organoid. (b, e) Statistically significant differences were calculated by unpaired student's *t* test and *p* < 0.01 were selected. Lectins were categorized based on their binding specificities. Data are shown with *t* values. (c, f) Volcano plot with fold change and *p* values on *x*- and *y*-axes, respectively. The vertical line represents a fold-change cutoff of ≥2.0.

These lectins and glycosyltransferases changes were also similarly compared in organoids derived from the same patients (Figure 5). In addition, lectin microarray and RNA-seq data were also compared between normal pancreatic ducts/cancer (Case 1) and IPMN/cancer (Case 2). In both cases, the reactivity of AAL and rAAL was significantly elevated in the mutants, with *FUT6* and *FUT3* elevated in both cases. The binding intensity of the mannose-binding lectins (rRSL, rBC2LA, and rPAIIL) and galactose-binding lectins (rGal3C, rCGL2), plus expression of their respective responsive genes, were also compared but only inconsistent results were obtained (data not shown).

## 4. Discussion

A comprehensive analysis of glycan expression in 16 organoids derived from 14 pancreatic cancer patients showed that the reactivity of fucose-binding lectins (AAL, AOL) was higher in 13 KRAS mutant organoids compared to 3 wild-type organoids. Furthermore, we were able to analyze wild-type versus mutant characteristics in samples from the same patients, precluding arguments that differing fucosylation may be due to individual glycosylation statuses independent of KRAS [25]. In these 2 unique cases, elevated fucosylation was found in only the KRAS mutants, supporting our hypothesis that the cell surface glycans of KRAS mutant PDAC organoids are hyper-fucosylated.

Hyper-fucosylation is reported to associate with diverse functions, including regulation of inflammatory responses, signal transduction, cell proliferation, transcription, and adhesion [26]. Such machinery may also promote higher expression of abnormal fucosylation during carcinogenesis and tumor progression [27, 28]. As for the diagnostic detection of altered fucosylation, the CA-19-9 (sialyl lewis a) antigen, is a frequently used biomarker for PDAC and emphasizes the role of this posttranslational modification on cancer adaptation and progression [4, 29, 30].

As the responsible gene for fucosylation, fucosyltransfereases such as *FUT6* mediate *α*1-3 fucosylation while *FUT3* mediates *α*1-3/4 fucosylation [26], playing a role as key enzymes for sialyl-Lewis X and/or CA19-9 generation [1, 31–34]. Our data demonstrated that KRAS mutants were hyper-*α*1-3/4 fucosylated and, at the same time, *FUT6* and *FUT3* RNA expression was actually elevated in our KRAS mutant organoids. Although we did not obtain direct mechanistic evidence for this hyper-fucosylation regarding KRAS, a study by Kakuma et al. indicates that EGF may be important [35]. They reported that, when EGF was used in colon cancer cell lines, glycosyltransferase, including *FUT3*, *FUT6*, and *ST3GAL1/3/4*, was elevated. Since EGF is the upstream molecule of the RAS cascade, the administration of EGF is considered synonymous with RAS mutation through activation of the RAS signaling pathway. In addition, another report demonstrated that RAS oncogenes upregulate glycosyltransferases, namely, *MGAT5*, via Ets-1 gene expression [36], and RAS oncogenes also upregulate *ST6GAL1* expression via RalGEF signaling [37]. Those studies support our idea that KRAS mutations are associated with increased expression of *FUT6/3*, resulting in increased *α*1-3/4-fucosylation in PDAC. Based on those reports, we regard it as reasonable that hyper-fucosylation of PDAC surface glycans may be mediated by KRAS mutation.

We also found that reactivity to mannose-binding lectins was increased and reactivity to galactose-binding lectins was decreased in KRAS mutant organoids. Mannose glycans that bind rRSL and rBC2LCA lectins have been shown to have anticancer and anti-inflammatory effects by promoting TGF-*β* activation and Treg cell induction [38, 39]. In other types of cancer, a high abundance of mannose glycans has been reported in the liver, ovaries, breasts, and lungs, but their presence in PDAC is largely unknown, and further studies are needed [40–43].

Another finding was that galactose-binding lectins, such as rGal3C and rCGL2, were decreased in KRAS mutant organoids. These galactose-binding lectins are soluble immunomodulatory proteins that have roles in intercellular adhesion, cell-matrix adhesion, and the modulation of cell surface receptor signaling efficiency [44]. Furthermore, as the carbohydrate-binding specificity of each galactose-binding lectin is intricately governed by sulfation, sialylation, fucosylation, and repeating N-acetyllactosamine units [45], the increased galectin binding to KRAS wild-type organoids that we observed may rely on these complex mechanisms and therefore warrant further investigation.

We further investigated whether the site of the KRAS mutation affects fucose glycans by comparing the KRAS G12V mutation group (7 cases) with the KRASG12D mutation group (5 cases). Both AAL and rAAL binding were significantly increased in the KRAS G12V mutant group but there were no significant differences in *FUT6* expression between the two groups. As different KRAS subtypes are known to cause various changes in signal activation [46], different glycans may be expressed depending on the KRAS mutation site. Further investigation is needed to understand the molecular mechanism of glycan changes caused by KRAS mutation sites.

We demonstrated the importance of the reactivity of AAL and AOL lectins to PDAC. Since AAL lectin has been shown to have a higher binding affinity to the surface of pancreatic cancer cells than the CA19-9 antibody, it may be possible to use those lectins as a novel therapeutic carrier and diagnostic tool for pancreatic cancer [47–49]. Another interesting plan derived from our results is to analyze the relationship between KRAS mutations and serum CA19-9, as it is well reported that CA19-9 does not substantially increase in proportion within a PDAC patient's serum even with positive Lewis antigen [50]. If our interpretation that the KRAS mutation causes an elevation in *FUT*3/6, in turn raising CA-19-9, is correct, then the PDAC tumors from serum CA19-9-negative patients may also be KRAS mutation-negative. However, it should be noted that, in Lewis-negative patients, *FUT*3 is defective, and thus CA19-9 expression would not be observed regardless of KRAS mutation status.

Two of the 14 PDAC organoids were established from patients who received preoperative chemotherapy. As it is commonly accepted that chemotherapies tend to be more effective against proliferative subpopulation which may bias the established organoid lines toward the more quiescence subpopulation that may display a very distinct phenotype with regard to FUT3/6 expression and lectin reactivity; we repeated the analyses, segregating samples from patients receiving prior neoadjuvant therapy from those that did not.

FUT3/6 mRNA levels were not significantly different between the preoperative chemotherapy and no treatment groups, and both were higher compared to the KRAS wild-type group. The gene expression data suggested that neoadjuvant therapy did not impact the phenotype of the established organoid lines (Figure S2(c)).

However, AAL, rAAL, AOL, and rAOL lectin intensities were lower in the preoperative chemotherapy group than in the no-treatment group and were not significantly different from those in the KRAS wild-type group, suggesting that neoadjuvant therapy may preferentially deplete the population that displayed high reactivity toward AAL and AOL despite retaining higher expression of FUT3/6 mRNA. Although our bulk data cannot discern whether there is a spectrum of AAL and AOL reactivity among the KRAS-mutated FUT3/6 high cells and the small sample size of this study precludes a robust conclusion from being drawn, this observation warrants further studies. For one, it may lead to the understanding of the link between AAL and AOL reactivity and sensitivity to chemotherapy. Moreover, since these cells retain higher FUT3/6 expression, it may be possible to devise a combinatorial therapy that first pushes the KRAS mutated, high FUT3/6 expression to the AAL and AOL high phenotypic states that are more sensitive toward chemotherapy.

Reports focusing on glycan conformational changes associated with KRAS mutations are scarce in pancreatic cancer, and this is the first report using organoids to analyze glycan changes associated with KRAS mutations. The identification of lectins that bind predominantly to KRAS mutant organoids compared to KRAS wild-type organoids may contribute to the development of markers that recognize KRAS mutant pancreatic cancer cells and cancer proteins for CTC and hematological diagnosis. Furthermore, the high affinity of lectins for binding specific targets on the outermost layers of cells underscores their potential as drug carriers [51]. The AAL and AOL lectins in this study have the potential to selectively avoid KRAS wild-type binding, facilitating the development of specific drug carriers that may suppress off-target cell damage.

We must acknowledge two main limitations of this study. First, organoids are known to resemble primary tumors in genetic and protein expression, a fact also exploited in obstetric medicine. However, since stromal and inflammatory cells undergo complex interactions with glycan structures in tumors, the present results may not be completely congruent with clinical samples. Therefore, to further validate the results of this study for clinical practice, single-cell analyses of the relationship between KRAS mutations, glycosyltransferases, and lectins will be necessary in the future.

Second, in this study, we focused only on the identification of glycosyltransferases and corresponding lectins affected by KRAS mutations. Further functional evaluation of glycosyltransferases in wild-type and KRAS mutant organoids and cell lines will require overexpression/knock-down cloning of glycosyltransferases and their functional evaluation.

## 5. Conclusion

Taken together, our data assert that KRAS mutations in PDAC increase *FUT6/3* expression and may contribute to the increased reactivity of the fucose-binding lectins AAL and AOL. These results support the potential utility of AAL lectin as a diagnostic marker for PDAC and as a potential target for the development of novel anticancer drugs, suggesting an association between KRAS mutations and fucosylation.

## Data Availability

The RNA-seq data used to support the findings of this study are available from the corresponding author upon request. And the lectin microarray data used to support the findings of this study are included within the supplementary information files.

## Abbreviations

PDAC: Pancreatic ductal adenocarcinoma
IPMN: Intraductal papillary mucinous neoplasm
PCA: Principal component analysis
SSPPD: Subtotal stomach preserving pancreaticoduodenectomy
DP: Distal pancreatectomy.

## Supplementary Materials

Supplementary Materials Figure S1: Brightfield images representative of organoid cultures (upper: KRAS wild-type, lower: KRAS mutant) in medium with EGF removed at day 5. Figure S2: Analysis of lectin intensity and FUT6 expression by site of KRAS mutation. Data are shown as means ± SD. Student's *t*-test. ^*∗∗*^*p* < 0.001. ^*∗*^*p* < 0.05. (a) Reactivity of fucose-binding lectin signal intensities from lectin microarray for KRAS wild -type (WT), Q61L, G12V, and G12D(a-1). Comparison of the intensities with WT and G12V, WT and G12D, respectively (a-2). (b) Expression of the FUT6 mRNA from RNA seq for KRAS wild -type (WT), Q61L, G12V, and G12D (b-1). Comparison of the expression with WT and G12V, WT and G12D, respectively (b-2). (c) FUT6 mRNA expression from RNA seq and reactivity of fucose-binding lectin signal intensities from lectin microarray of organoids from KRAS wild-type (WT) and preoperative treated (Neoadjuvant) and untreated (No treat) patients. Figure S3: Comparison of glycosyltransferases (mannnosyltransferase, galactosyltransferase, sialyltransferase) expression based on the result of RNA-seq between KRAS mutant and KRAS wild-type organoids. Table S1: Clinical information for the organoids. Table S2: Lectin signals in KRAS mutant (MT) and KRAS wild-type (WT) organoids.

## References

[1] Pinho S. S., Reis C. A. (2015). Glycosylation in cancer: mechanisms and clinical implications. *Nature Reviews Cancer*.

[2] Ohtsubo K., Marth J. D. (2006). Glycosylation in cellular mechanisms of health and disease. *Cell*.

[3] Hart G. W., Copeland R. J. (2010). Glycomics hits the big time. *Cell*.

[4] Lumibao J. C., Tremblay J. R., Hsu J., Engle D. D. (2022). Altered glycosylation in pancreatic cancer and beyond. *Journal of Experimental Medicine*.

[5] Ryan D. P., Hong T. S., Bardeesy N. (2014). Pancreatic adenocarcinoma. *New England Journal of Medicine*.

[6] Kamisawa T., Wood L. D., Itoi T., Takaori K. (2016). Pancreatic cancer. *The Lancet*.

[7] Waters A. M., Der C. J. (2018). KRAS: the critical driver and therapeutic target for pancreatic cancer. *Cold Spring Harb Perspect Med*.

[8] Kanda M., Matthaei H., Wu J. (2012). Presence of somatic mutations in most early-stage pancreatic intraepithelial neoplasia. *Gastroenterology*.

[9] Liu J., Ji S., Liang C. (2016). Critical role of oncogenic KRAS in pancreatic cancer (Review). *Molecular Medicine Reports*.

[10] Bannoura S. F., Uddin M. H., Nagasaka M. (2021). Targeting KRAS in pancreatic cancer: new drugs on the horizon. *Cancer and Metastasis Reviews*.

[11] Wagatsuma T., Nagai-Okatani C., Matsuda A. (2020). Discovery of pancreatic ductal adenocarcinoma-related aberrant glycosylations: a multilateral approach of lectin microarray-based tissue glycomic profiling with public transcriptomic datasets. *Frontiers Oncology*.

[12] Orth M., Metzger P., Gerum S. (2019). Pancreatic ductal adenocarcinoma: biological hallmarks, current status, and future perspectives of combined modality treatment approaches. *Radiation Oncology*.

[13] van de Wetering M., Francies H. E., Francis J. M. (2015). Prospective derivation of a living organoid biobank of colorectal cancer patients. *Cell*.

[14] Tuveson D., Clevers H. (2019). Cancer modeling meets human organoid technology. *Science*.

[15] Rossi G., Manfrin A., Lutolf M. P. (2018). Progress and potential in organoid research. *Nature Reviews Genetics*.

[16] Moreira L., Bakir B., Chatterji P., Dantes Z., Reichert M., Rustgi A. K. (2018). Pancreas 3D organoids: current and future aspects as a research platform for personalized medicine in pancreatic cancer. *Cellular and Molecular Gastroenterology and Hepatology*.

[17] Seino T., Kawasaki S., Shimokawa M. (2018). Human pancreatic tumor organoids reveal loss of stem cell niche factor dependence during disease progression. *Cell Stem Cell*.

[18] Mihara E., Hirai H., Yamamoto H. (2016). Active and water-soluble form of lipidated Wnt protein is maintained by a serum glycoprotein afamin/*α*-albumin. *Elife*.

[19] Fujii M., Sato T. (2021). Somatic cell-derived organoids as prototypes of human epithelial tissues and diseases. *Nature Materials*.

[20] Tateno H., Toyota M., Saito S. (2011). Glycome diagnosis of human induced pluripotent stem cells using lectin microarray. *Journal of Biological Chemistry*.

[21] Dobin A., Davis C. A., Schlesinger F. (2013). STAR: ultrafast universal RNA-seq aligner. *Bioinformatics*.

[22] Oshlack A., Robinson M. D., Young M. D. (2010). From RNA-seq reads to differential expression results. *Genome Biology*.

[23] Robinson J. T., Thorvaldsdóttir H., Winckler W. (2011). Integrative genomics viewer. *Nature Biotechnology*.

[24] Vajaria B. N., Patel P. S. (2017). Glycosylation: a hallmark of cancer?. *Glycoconjugate Journal*.

[25] Knezević A., Polasek O., Gornik O. (2009). Variability, heritability and environmental determinants of human plasma N-glycome. *Journal of Proteome Research*.

[26] Becker D. J., Lowe J. B. (2003). Fucose: biosynthesis and biological function in mammals. *Glycobiology*.

[27] Vanhooren P. T., Vandamme E. J. (1999). L-Fucose: occurrence, physiological role, chemical, enzymatic and microbial synthesis. *Journal of Chemical Technology and Biotechnology*.

[28] Peixoto A., Relvas-Santos M., Azevedo R., Santos L. L., Ferreira J. A. (2019). Protein glycosylation and tumor microenvironment alterations driving cancer hallmarks. *Frontiers Oncology*.

[29] Yue T., Partyka K., Maupin K. A. (2011). Identification of blood-protein carriers of the CA 19-9 antigen and characterization of prevalence in pancreatic diseases. *Proteomics*.

[30] Mereiter S., Balmana M., Campos D., Gomes J., Reis C. A. (2019). Glycosylation in the era of cancer-targeted therapy: where are we heading?. *Cancer Cell*.

[31] Blanas A., Sahasrabudhe N. M., Rodriguez E., van Kooyk Y., van Vliet S. J. (2018). Fucosylated antigens in cancer: an alliance toward tumor progression, metastasis, and resistance to chemotherapy. *Frontiers Oncology*.

[32] He M., Wu C., Xu J. (2014). A genome wide association study of genetic loci that influence tumour biomarkers cancer antigen 19-9, carcinoembryonic antigen and *α* fetoprotein and their associations with cancer risk. *Gut*.

[33] Dohi T., Hashiguchi M., Yamamoto S., Morita H., Oshima M. (1994). Fucosyltransferase-producing sialyl Le(a) and sialyl Le(x) carbohydrate antigen in benign and malignant gastrointestinal mucosa. *Cancer*.

[34] Luo G., Jin K., Deng S. (2021). Roles of CA19-9 in pancreatic cancer: biomarker, predictor and promoter. *Biochimica et Biophysica Acta, Reviews on Cancer*.

[35] Sakuma K., Aoki M., Kannagi R. (2012). Transcription factors c-Myc and CDX2 mediate E-selectin ligand expression in colon cancer cells undergoing EGF/bFGF-induced epithelial-mesenchymal transition. *Proceedings of the National Academy of Sciences of the U S A*.

[36] Wojciechowicz D. C., Park P. Y., Datta R. V., Paty P. B. (2000). CEA is the major PHA-L-reactive glycoprotein in colon carcinoma cell lines and tumors: relationship between K-ras activation and *β*1–6 branching of N-linked carbohydrate on CEA. *Biochemical and Biophysical Research Communications*.

[37] Dalziel M., Dall’Olio F., Mungul A., Piller V., Piller F. (2004). Ras oncogene induces *β*‐galactoside *α*2,6‐sialyltransferase (ST6Gal I) via a RalGEF‐mediated signal to its housekeeping promoter. *European Journal of Biochemistry*.

[38] Zhang D., Chia C., Jiao X. (2017). D-mannose induces regulatory T cells and suppresses immunopathology. *Nature Medicine*.

[39] Torretta S., Scagliola A., Ricci L. (2020). D-mannose suppresses macrophage IL-1*β* production. *Nature Communications*.

[40] Takayama H., Ohta M., Iwashita Y. (2020). Altered glycosylation associated with dedifferentiation of hepatocellular carcinoma: a lectin microarray-based study. *Bone Marrow Concentrate Cancer*.

[41] Everest-Dass A. V., Briggs M. T., Kaur G., Oehler M. K., Hoffmann P., Packer N. H. (2016). N-Glycan MALDI imaging mass spectrometry on formalin-fixed paraffin-embedded tissue enables the delineation of ovarian cancer tissues. *Molecular & Cellular Proteomics*.

[42] Scupakova K., Adelaja O. T., Balluff B. (2021). Clinical importance of high-mannose, fucosylated, and complex N-glycans in breast cancer metastasis. *Journal of Clinical Investigation Insight*.

[43] Ruhaak L. R., Taylor S. L., Stroble C. (2015). Differential N-glycosylation patterns in lung adenocarcinoma tissue. *Journal of Proteome Research*.

[44] Liu F. T., Rabinovich G. A. (2005). Galectins as modulators of tumour progression. *Nature Reviews Cancer*.

[45] Dimitroff C. J. (2015). Galectin-binding O-glycosylations as regulators of malignancy. *Cancer Research*.

[46] Munoz-Maldonado C., Zimmer Y., Medova M. (2019). A comparative analysis of individual RAS mutations in cancer biology. *Frontiers Oncology*.

[47] Nie S., Lo A., Wu J. (2014). Glycoprotein biomarker panel for pancreatic cancer discovered by quantitative proteomics analysis. *Journal of Proteome Research*.

[48] Kim S., Choi Y., Kim K. (2022). Coacervate-mediated novel pancreatic cancer drug Aleuria Aurantia lectin delivery for augmented anticancer therapy. *Biomaterials Research*.

[49] Choi Y., Park U., Koo H. J. (2021). Exosome-mediated diagnosis of pancreatic cancer using lectin-conjugated nanoparticles bound to selective glycans. *Biosensors and Bioelectronics*.

[50] Chan A., Prassas I., Dimitromanolakis A. (2014). Validation of biomarkers that complement CA19.9 in detecting early pancreatic cancer. *Clinical Cancer Research*.

[51] Shimomura O., Oda T., Tateno H. (2018). A novel therapeutic strategy for pancreatic cancer: targeting cell surface glycan using rBC2LC-N lectin-drug conjugate (LDC). *Molecular Cancer Therapeutics*.

