# Supplementary material for: Aberrant Glycosylation in Pancreatic Ductal Adenocarcinoma 3D Organoids Is Mediated by KRAS Mutations
Source: J Oncol. 2024 Mar 18;2024:1529449. doi: 10.1155/2024/1529449 (PMC10963106; doi:10.1155/2024/1529449)
Supplement: Supplementary Materials — Figure S1: Brightfield images representative of organoid cultures (upper: KRAS wild-type, lower: KRAS mutant) in medium with EGF removed at day 5. Figure S2: Analysis of lectin intensity and FUT6 expression by site of KRAS mutation. Data are shown as means ± SD. Student's t-test. ∗∗p < 0.001. ∗p < 0.05. (a) Reactivity of fucose-binding lectin signal intensities from lectin microarray for KRAS wild -type (WT), Q61L, G12V, and G12D(a-1). Comparison of the intensities with WT and G12V, WT and G12D, respectively (a-2). (b) Expression of the FUT6 mRNA from RNA seq for KRAS wild -type (WT), Q61L, G12V, and G12D (b-1). Comparison of the expression with WT and G12V, WT and G12D, respectively (b-2). (c) FUT6 mRNA expression from RNA seq and reactivity of fucose-binding lectin signal intensities from lectin microarray of organoids from KRAS wild-type (WT) and preoperative treated (Neoadjuvant) and untreated (No treat) patients. Figure S3: Comparison of glycosyltransferases (mannnosyltransferase, galactosyltransferase, sialyltransferase) expression based on the result of RNA-seq between KRAS mutant and KRAS wild-type organoids. Table S1: Clinical information for the organoids. Table S2: Lectin signals in KRAS mutant (MT) and KRAS wild-type (WT) organoids. [file 1529449.f1.zip › Supplementary Figure.pdf]

Supplementary Fig1

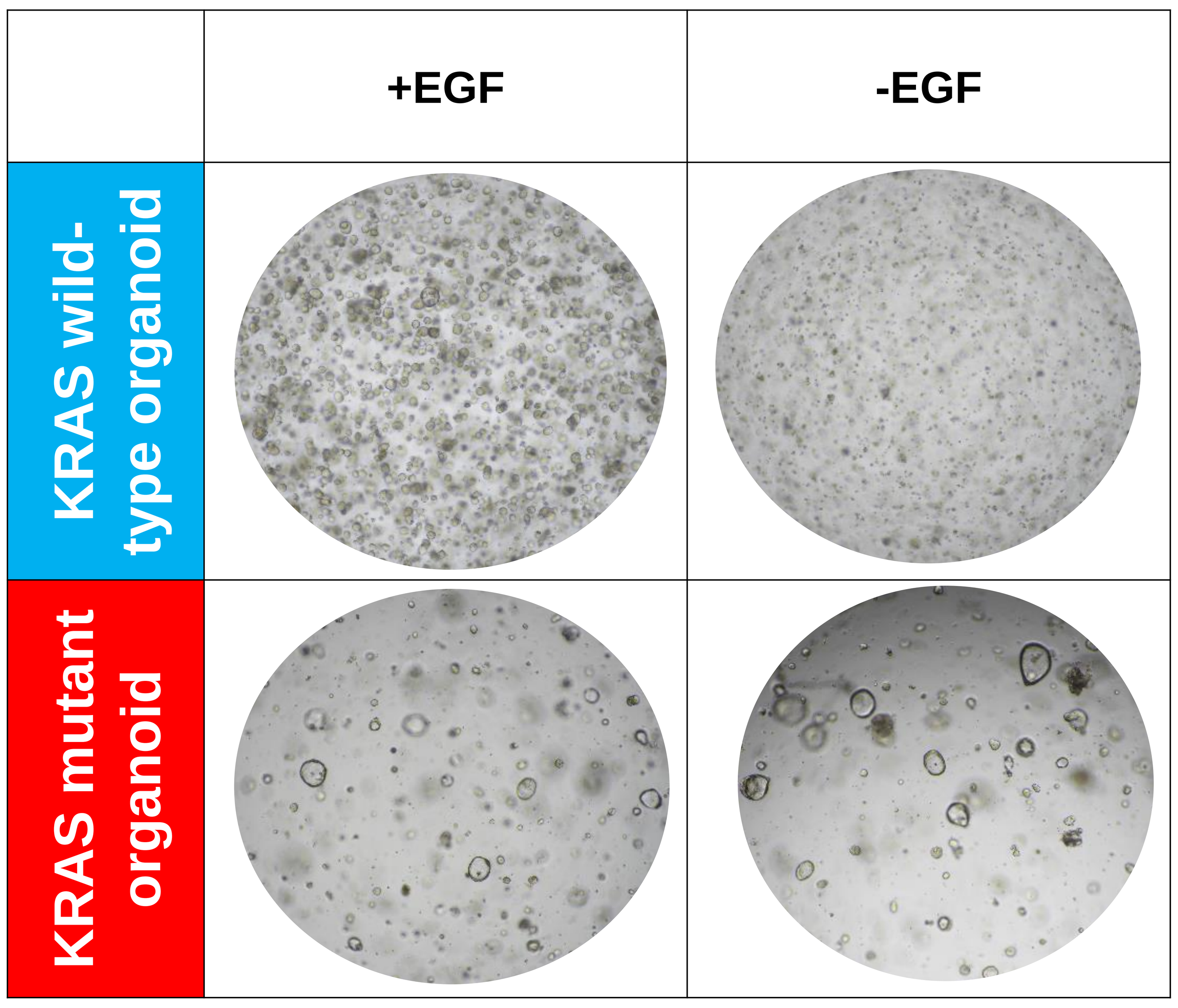

Figure S1: Brightfield images representative of organoid cultures (upper: KRAS wild-type, lower: KRAS mutant) in medium with EGF removed at day 5.

Supplementary Fig2

a Fucose binding Lectin

1)

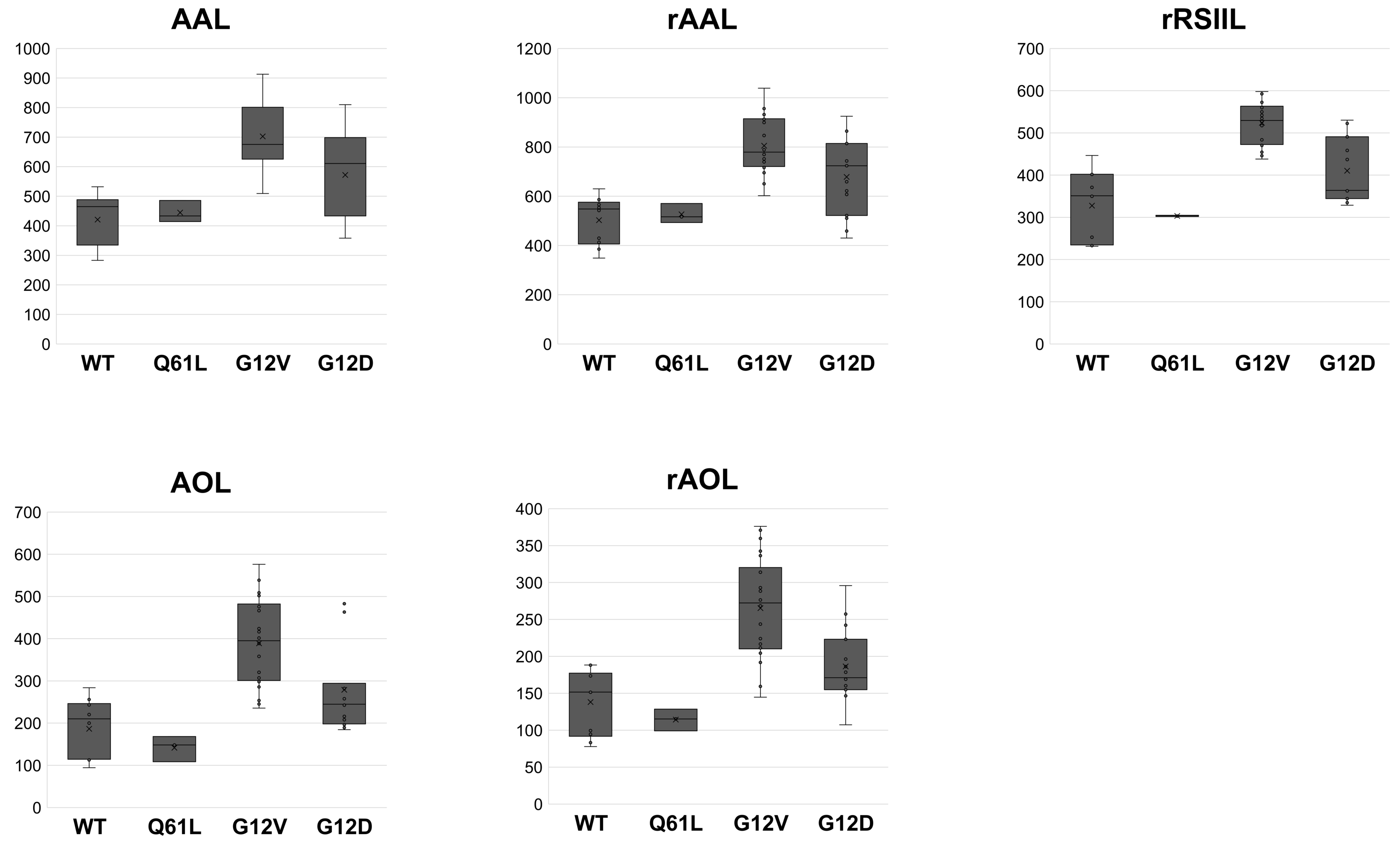

2)

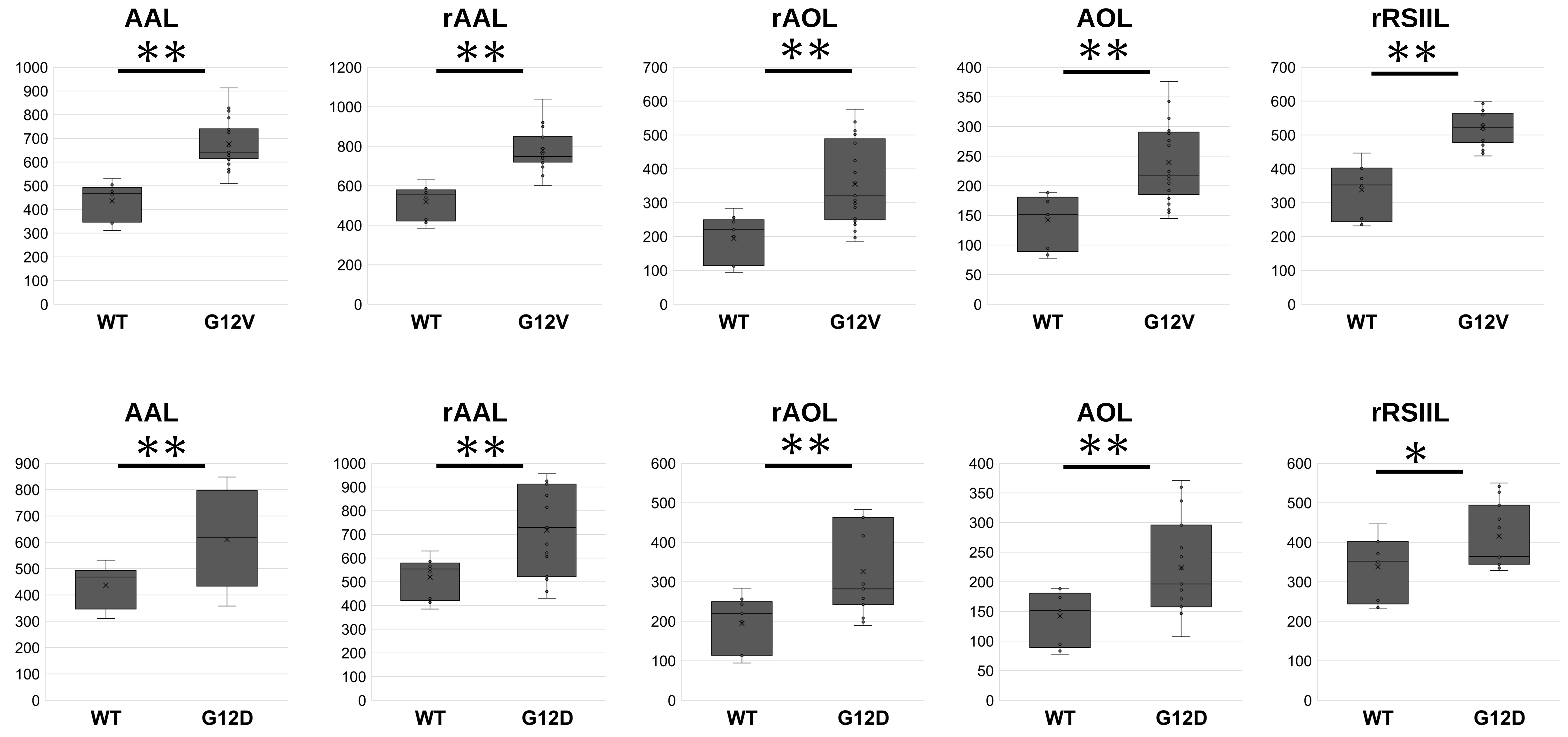

Supplementary Fig2

b Fucosyltransferase

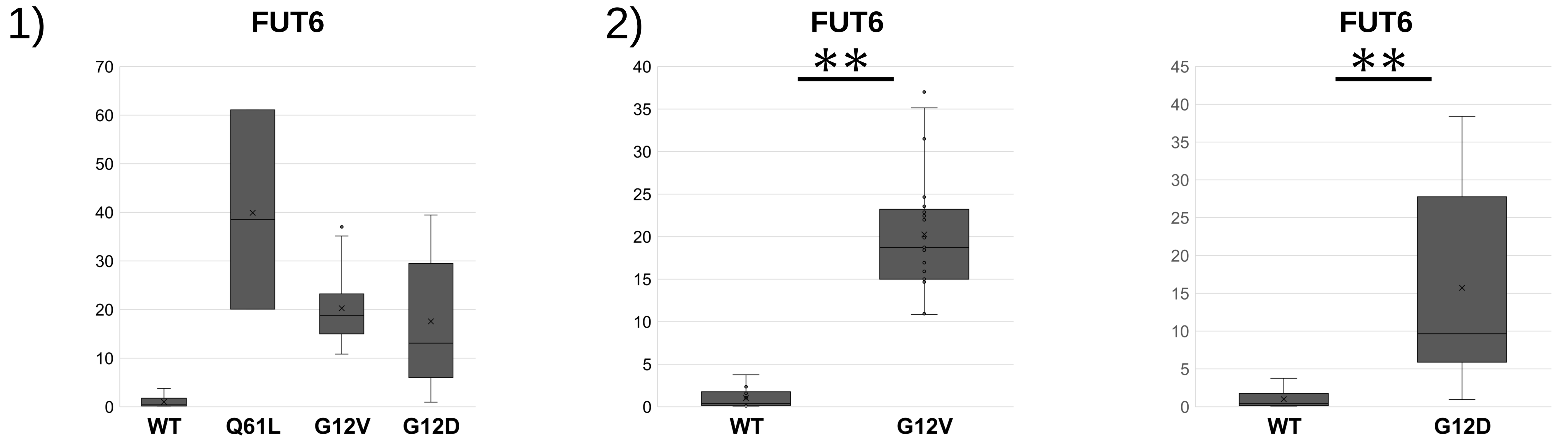

c

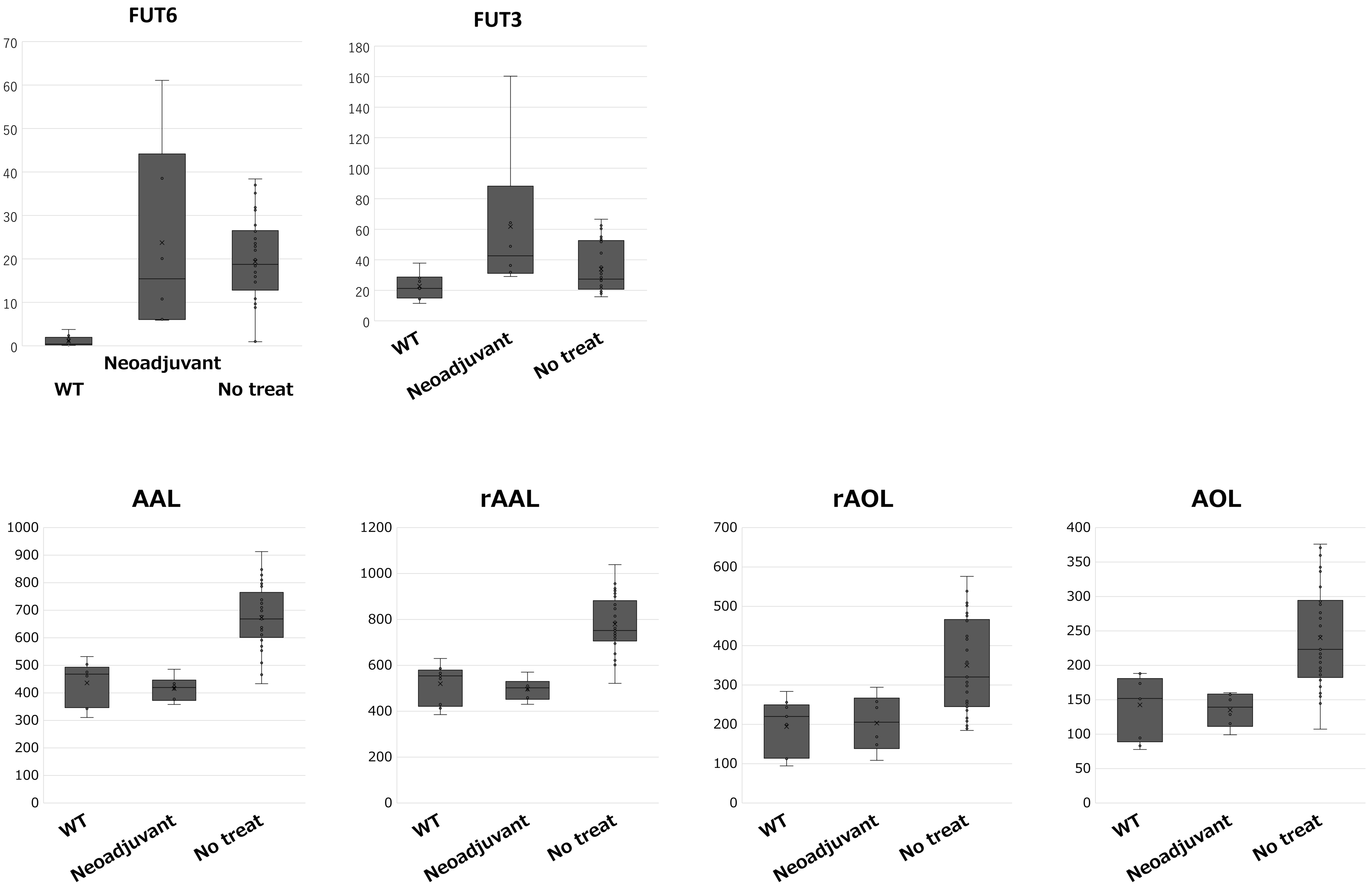

Fig S2: Analysis of lectin intensity and FUT6 expression by site of KRAS mutation

Data are shown as means ±SD. Student's t test. **p < 0.001. *p < 0.05.

a) Reactivity of fucose-binding lectin signal intensities from lectin microarray for KRAS wild -type (WT), Q61L, G12V, and G12D(a-1). Comparison of the intensities with WT and G12V, WT and G12D, respectively (a-2).

b) Expression of the FUT6 mRNA from RNA seq for KRAS wild -type (WT), Q61L, G12V, and G12D (b-1). Comparison of the expression with WT and G12V, WT and G12D, respectively (b-2).

c) FUT6 mRNA expression from RNA seq and reactivity of fucose-binding lectin signal intensities from lectin microarray of organoids from KRAS wild-type (WT) and preoperative treated (Neoadjuvant) and untreated (No treat) patients.

Supplementary Fig3

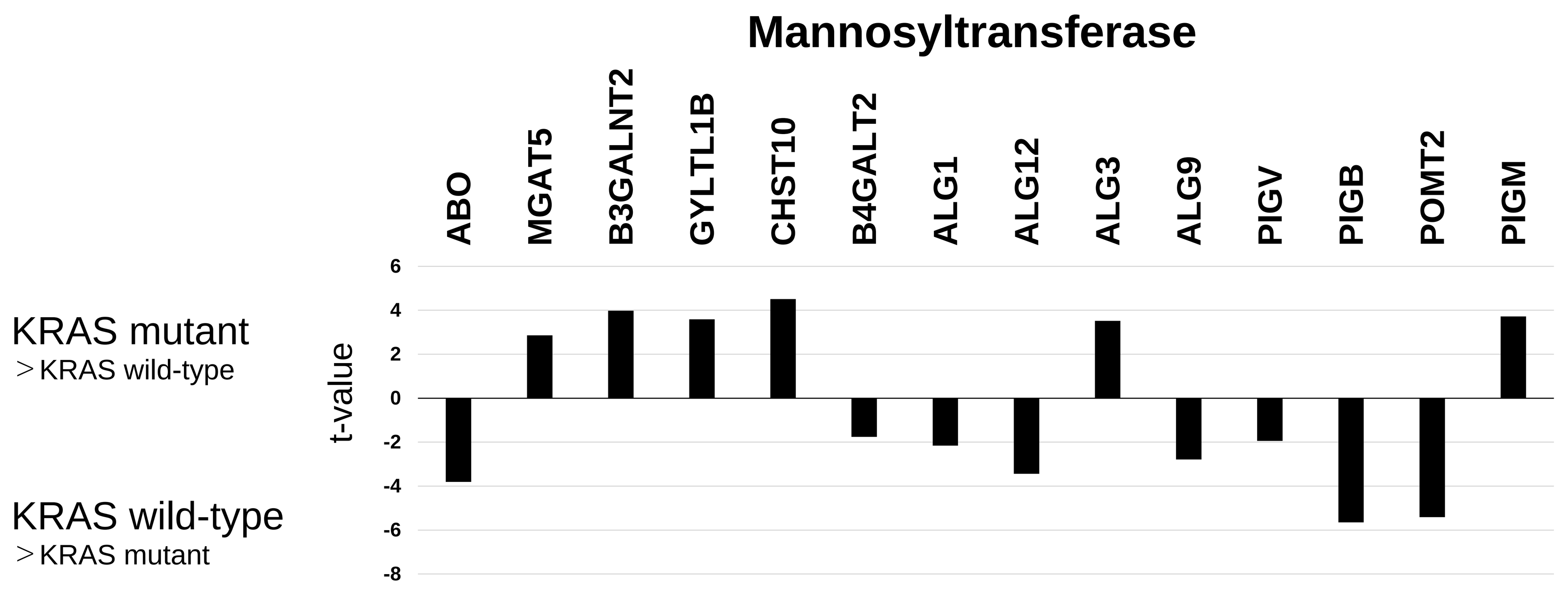

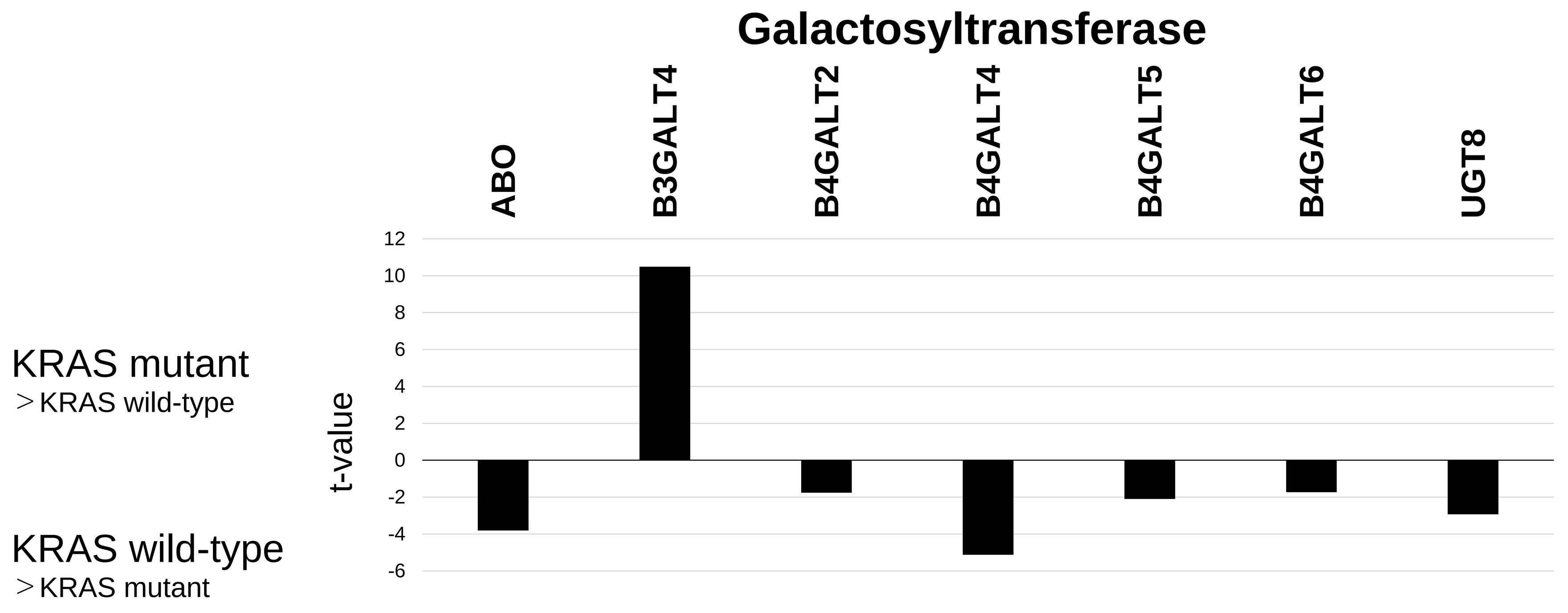

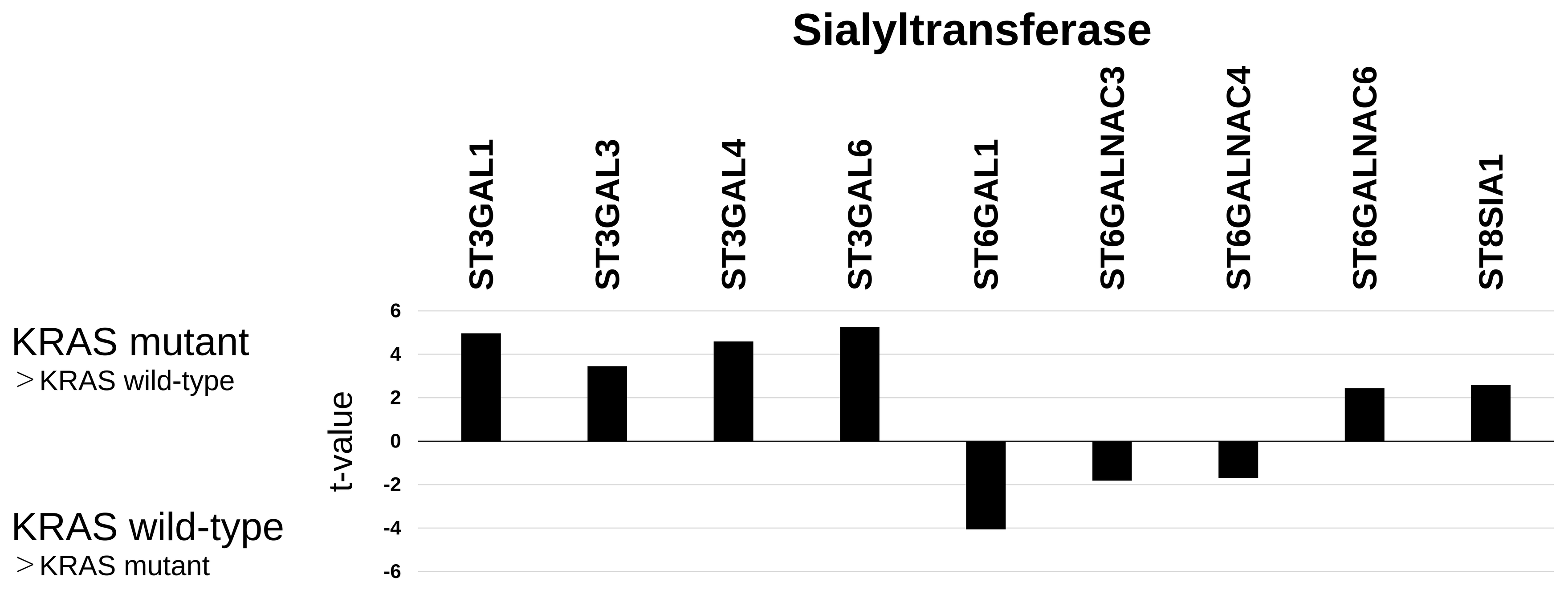

Figure S3: Comparison of glycosyltransferases (mannnosyltransferase, galactosyltransferase, sialyltransferase) expression based on the result of RNA-seq between KRAS mutant and KRAS wild-type organoids
