# Supplementary material for: Aberrant Glycosylation in Pancreatic Ductal Adenocarcinoma 3D Organoids Is Mediated by KRAS Mutations
Source: J Oncol. 2024 Mar 18;2024:1529449. doi: 10.1155/2024/1529449 (PMC10963106; doi:10.1155/2024/1529449)
Supplement: Supplementary Materials — Figure S1: Brightfield images representative of organoid cultures (upper: KRAS wild-type, lower: KRAS mutant) in medium with EGF removed at day 5. Figure S2: Analysis of lectin intensity and FUT6 expression by site of KRAS mutation. Data are shown as means ± SD. Student's t-test. ∗∗p < 0.001. ∗p < 0.05. (a) Reactivity of fucose-binding lectin signal intensities from lectin microarray for KRAS wild -type (WT), Q61L, G12V, and G12D(a-1). Comparison of the intensities with WT and G12V, WT and G12D, respectively (a-2). (b) Expression of the FUT6 mRNA from RNA seq for KRAS wild -type (WT), Q61L, G12V, and G12D (b-1). Comparison of the expression with WT and G12V, WT and G12D, respectively (b-2). (c) FUT6 mRNA expression from RNA seq and reactivity of fucose-binding lectin signal intensities from lectin microarray of organoids from KRAS wild-type (WT) and preoperative treated (Neoadjuvant) and untreated (No treat) patients. Figure S3: Comparison of glycosyltransferases (mannnosyltransferase, galactosyltransferase, sialyltransferase) expression based on the result of RNA-seq between KRAS mutant and KRAS wild-type organoids. Table S1: Clinical information for the organoids. Table S2: Lectin signals in KRAS mutant (MT) and KRAS wild-type (WT) organoids. [file 1529449.f1.zip › Supplementary Tables.docx]

**Table S1:** Clinical information for the organoids

| No. | Age | Gender | Location | Procedure | Adjuvant Chemotherapy | Histology | UICC | pT | pN | pM |
| --- | --- | --- | --- | --- | --- | --- | --- | --- | --- | --- |
| PCO1 | 77 | F | tale | Total pancreatectomy | TS-1 | mod＞well | IB | 3 | 0 | 0 |
| PCO2 | 66 | M | head | SSPPD | 0 | mod | Ⅲ | 3 | 2 | 0 |
| PCO3 | 75 | M | head | SSPPD | 0 | mod＞por | Ⅲ | 3 | 1b | 0 |
| PCO4 | 69 | M | head | SSPPD | 0 | mod | Ⅲ | 3 | 1b | 0 |
| PCO5 | 66 | F | head | SSPPD | 0 | mod | Ⅲ | 3 | 1b | 0 |
| PCO6 | 67 | F | head | SSPPD | 0 | mod | Ⅲ | 3 | 1b | 0 |
| PCO7 | 70 | M | head | Total pancreatectomy | 0 | mod＞wel＞por | ⅡB | 2 | 1a | 0 |
| PCO8 | 68 | F | body | DP | GEM+nabPTX | mod | ⅡB | 3 | 1a | 0 |
| PCO9 | 67 | F | tale | DP | GEM+Radiation | well/mod | ⅠB | 3 | 0 | 0 |
| PCO10 | 68 | M | head | SSPPD | 0 | well | ⅠB | 3 | 0 | 0 |
| PCO11 | 68 | M | body | DP | 0 | mod | ⅠA | 1c | 0 | 0 |
| PCO12 | 64 | F | head | SSPPD | 0 | well | Ⅲ | 3 | 1b | 0 |
| PCO13 | 76 | M | head | SSPPD | 0 | well | ⅠB | 3 | 0 | 0 |
| PCO14 | 58 | M | head | SSPPD | 0 | mod | Ⅲ | 3 | 1b | 0 |
| PCO15 | 54 | M | body | SSPPD | 0 | mod | Ⅲ | 3 | 1b | 0 |
| PCO16 | 84 | F | body | DP | 0 | mod＞por | ⅡA | 3 | 0 | 0 |
| PCO17 | 73 | M | tale | DP | TS-1, GC | mod＞well＞por | ⅡB | 3 | 1a | 0 |
| PCO18 | 55 | F | head | SSPPD | 0 | por | Ⅲ | 3 | 1b | 0 |
| PCO19 | 82 | M | head | SSPPD | 0 | mod | Ⅲ | 3 | 1b | 0 |
| PCO20 | 80 | M | head | SSPPD | 0 | mod | ⅠB | 3 | 0 | 0 |
| PCO21 | 72 | M | body | DP | 0 | mod | ⅡB | 3 | 1a | 0 |
| PCO22 | 68 | F | head | SSPPD | 0 | mod | ⅡB | 3 | 1a | 0 |
| PCO23 | 82 | M | head | SSSPD | 0 | mod | ⅡB | 3 | 1a | 0 |
| PCO24 | 67 | F | head | SSPPD | 0 | mod | ⅡB | 3 | 1a | 0 |

**Table S2:** Lectin signals in KRAS mutant (MT) and KRAS wild-type (WT) organoids

|  | species | WT | |  | MT | | *p*-value | Rough specificity^1,2^ |
| --- | --- | --- | --- | --- | --- | --- | --- | --- |
|  |  | mean | SEM |  | mean | SEM |  |  |
| LFA | *Limax flavus* | 10.4945 | 2.5621 |  | 13.1582 | 2.082 | 0.42817 | Sia |
| WGA | *Triticum unlgari* | 12.3768 | 5.1825 |  | 3.20067 | 0.823 | 0.11265 | (GlcNAc)n, polySia |
| PVL | *Psathyrella velutina* | 131.455 | 8.9588 |  | 146.468 | 5.346 | 0.169317 | Sia, GlcNAc |
| MAL | *Maackia amurensis* | 0 | 0 |  | 0 | 0 | - | α2-3Sia |
| MAH | *Maackia amurensis* | 0.48808 | 0.2979 |  | 0.74949 | 0.263 | 0.516627 | α2-3Sia |
| ACG | *Agrocybe cylindracea* | 0.79122 | 0.4309 |  | 0.64349 | 0.268 | 0.774542 | α2-3Sia |
| rACG | *Agrocybe cylindracea* (recombinant) | 88.1112 | 20.242 |  | 72.159 | 6.759 | 0.470309 | α2-3Sia |
| rGal8N | *Homo sapiens* (recombinant) | 36.9201 | 4.1567 |  | 51.7435 | 4.268 | 0.01858 | α2-3Sia |
| SNA | *Sambucus nigra* | 57.326 | 9.385 |  | 35.3182 | 4.786 | 0.055349 | α2-6Sia |
| SSA | *Sambucus sieboldiana* | 108.262 | 17.664 |  | 63.7903 | 7.297 | 0.037882 | α2-6Sia |
| TJAI | *Trichosanthes japonica* | 76.7214 | 14.353 |  | 47.5441 | 6.297 | 0.085986 | α2-6Sia |
| rPSL1a | *Polyporus squamosus* (recombinant) | 150.55 | 20.815 |  | 128.411 | 8.614 | 0.344704 | α2-6Sia |
| ADA | *Allomyrina dichtoma* | 66.3429 | 19.959 |  | 20.8764 | 7.284 | 0.054538 | α2-6Sia, Forssman, A, B |
| PHAL | *Phaseolus vulgaris* | 13.1713 | 2.918 |  | 6.21484 | 1.467 | 0.051511 | GlcNAcβ1-6Man (Tetraantenna) |
| DSA | *Datura stramonium* | 214.031 | 44.685 |  | 167.234 | 13.32 | 0.337816 | GlcNAcβ1-6Man (Tetraantenna) |
| TxLcI | *Tulipa gesneriana* | 0 | 0 |  | 0.57092 | 0.286 | 0.052944 | Galactosylated *N-*glycans up to triantenna |
| ECA | *Erythrina cristagalli* | 4.03418 | 1.1417 |  | 6.99971 | 1.504 | 0.124262 | βGal |
| RCA120 | *Ricinus communis* | 111.229 | 18.089 |  | 82.0144 | 5.071 | 0.149645 | βGal |
| rGal7 | *Homo sapiens* (recombinant) | 0.41486 | 0.1267 |  | 0.11599 | 0.055 | 0.050175 | chondroitin polymer |
| rGal9N | *Homo sapiens* (recombinant) | 492.869 | 35.39 |  | 426.771 | 25.4 | 0.145271 | polyLacNAc |
| rGal9C | *Homo sapiens* (recombinant) | 636.996 | 16.517 |  | 722.285 | 28.51 | 0.012805 | polyLacNAc, Branched LacNAc |
| rC14 | *Gallus gallus domesticus* (recombinant) | 22.947 | 4.3867 |  | 18.2828 | 2.459 | 0.368184 | Branched LacNAc |
| rDiscoidin II | *Dictyostelium discoideum* (recombinant) | 8.56961 | 1.7983 |  | 4.06217 | 0.857 | 0.040881 | LacNAc, Galb1-3GalNAc (T), GalNAc (Tn) |
| BPL | *Bauhinia purpurea alba* | 51.5112 | 11.629 |  | 88.821 | 12.66 | 0.037379 | Galβ1-3GlcNAc(GalNAc), α/βGalNAc |
| rCGL2 | *Homo sapiens* (recombinant) | 451.119 | 22.745 |  | 305.213 | 18.83 | 5.36E-05 | GalNAcα1-3Gal (A), polyLacNAc |
| PHAE | *Phaseolus vulgaris* | 79.4164 | 11.171 |  | 63.1697 | 5.68 | 0.215732 | bisecting GlcNAc |
| GSLII | *Griffonia simplicifolia* | 0.10515 | 0.0789 |  | 0.26651 | 0.11 | 0.239561 | GlcNAcβ1-4Man |
| rSRL | *Sclerotium rolfsii* (recombinant) | 74.4468 | 16.264 |  | 88.0903 | 6.741 | 0.453029 | agalacto *N-*glycan |
| UDA | *Urtica dioica* | 662.772 | 79.778 |  | 800.021 | 31.76 | 0.135926 | (GlcNAc)n |
| PWM | *Phytolacca americana* | 2.14359 | 0.6001 |  | 1.55395 | 0.31 | 0.397125 | (GlcNAc)n |
| rF17AG | *Escherichia coli* (recombinant) | 8.27462 | 2.3553 |  | 6.08575 | 1.62 | 0.453477 | GlcNAc |
| rGRFT | *Griffithia* sp. | 157.965 | 8.5125 |  | 147.6 | 8.811 | 0.404137 | Man |
| NPA | *Narcissus pseudonarcissus* | 59.639 | 4.7799 |  | 80.3364 | 6.65 | 0.01539 | Manα1-3Man |
| ConA | *Canavalia ensiformis* | 75.6721 | 6.0042 |  | 90.48 | 4.506 | 0.06219 | M3, Manα1-2Manα1-3(Manα1-6)Man, GlcNAcβ1-2Manα1-3(Manα1-6)Man |
| GNA | *Galanthus nivalis* | 26.7347 | 2.4924 |  | 31.2154 | 3.144 | 0.271057 | Manα1-3Man, Manα1-6Man |
| HHL | *Hippeastrum hybrid* | 3.09491 | 1.3049 |  | 4.5748 | 1.005 | 0.379094 | Manα1-3Man, Manα1-7Man |
| ASA | *Allium sativum* | 0.43513 | 0.3405 |  | 0.34432 | 0.119 | 0.805777 | Galβ1-4GlcNAcβ1-2Man |
| DBAI | *Dioscorea batatas* | 40.3496 | 2.9267 |  | 35.1858 | 3.35 | 0.253704 | High-Man |
| CCA | *Castanea crenata* | 53.9651 | 5.4922 |  | 64.3239 | 3.385 | 0.127236 | Galactosylated *N-*glycans up to triantenna |
| Heltuba | *Helianthus t uberosus* | 94.3596 | 8.1624 |  | 105.339 | 5.353 | 0.275727 | Manα1-3Man |
| rHeltuba | *Helianthus t uberosus* (recombinant) | 40.5988 | 4.4158 |  | 39.6905 | 3.05 | 0.867429 | Manα1-3Man |
| VVAII | *Vicia villosa* | 0.32842 | 0.1866 |  | 0.73548 | 0.455 | 0.412367 | Man, Agalacto |
| rOrysata | *Oryza sativa* (recombinant) | 46.8222 | 3.8591 |  | 48.2912 | 3.474 | 0.779517 | Manα1-3Man, High-Man, biantenna |
| rPALa | *Phlebodium aureum* (recombinant) | 96.7841 | 10.345 |  | 110.07 | 4.998 | 0.267548 | Man5, biantenna |
| rBanana | *Musa acuminata* (recombinant) | 124.645 | 8.609 |  | 130.353 | 5.954 | 0.591957 | Manα1-2Manα1-3(6)Man |
| rCalsepa | *Calystegia sepium* (recombinant) | 37.4842 | 3.9581 |  | 47.6861 | 3.257 | 0.058626 | Biantenna with bisecting GlcNAc |
| rRSL | *Ralstonia solanacearum* (recombinant) | 178.408 | 6.6742 |  | 255.905 | 14.61 | 1.46E-05 | αMan, α1-2Fuc (H), α1-3Fuc (Lex), α1-4Fuc (Lea) |
| rBC2LA | *Burkholderia cenocepacia* (recombinant) | 188.823 | 8.9326 |  | 236.033 | 8.559 | 0.000695 | αMan, High-Man |
| AOL | *Aspergillus oryzae* | 138.178 | 14.124 |  | 224.365 | 12.07 | 0.000104 | α1-2Fuc (H), α1-3Fuc (Lex), α1-4Fuc (Lea) |
| AAL | *Aleuria aurantia* | 420.973 | 28.321 |  | 634.108 | 22.41 | 6.33E-06 | α1-2Fuc (H), α1-3Fuc (Lex), α1-4Fuc (Lea) |
| rAAL | *Aleuria aurantia* (reconbinant) | 502.764 | 31.116 |  | 736.782 | 23.86 | 6.33E-06 | α1-2Fuc (H), α1-3Fuc (Lex), α1-4Fuc (Lea) |
| rPAIIL | *Pseudomonas aeruginosa* (recombinant) | 48.9821 | 6.5574 |  | 76.0273 | 3.971 | 0.002726 | αMan, α1-2Fuc (H), α1-3Fuc (Lex), α1-4Fuc (Lea) |
| rRSIIL | *Ralstonia solanacearum* (recombinant) | 327.692 | 25.981 |  | 464.788 | 14.5 | 0.000334 | α1-2Fuc (H), α1-3Fuc (Lex), α1-4Fuc (Lea) |
| rPTL | *Pholiota terrestris* (recombinant) | 312.525 | 17.041 |  | 306.314 | 11.24 | 0.764455 | α1-6Fuc |
| PSA | *Pisum sativum* | 41.2014 | 4.9001 |  | 34.7894 | 2.693 | 0.269491 | α1-6Fuc up to biantenna |
| LCA | *Lens culinaris* | 77.5774 | 8.1359 |  | 59.5877 | 4.045 | 0.068004 | α1-6Fuc up to biantenna |
| rAOL | *Aspergillus oryzae* (recombinant) | 186.537 | 22.075 |  | 329.301 | 19.61 | 5.61E-05 | Fuc α1-2Galβ1-3GlcNAc (GalNAc) |
| rBC2LCN | *Burkholderia cenocepacia* (recombinant) | 223.848 | 30.257 |  | 288.358 | 14.15 | 0.075176 | Fuc α1-2Galβ1-3GlcNAc (GalNAc) |
| LTL | *Lotus tetragonolobus* | 0.72359 | 0.1504 |  | 1.29024 | 0.348 | 0.141313 | Lex, Ley |
| UEAI | *Ulex europaeus* | 6.05273 | 1.6902 |  | 28.8566 | 7.201 | 0.00358 | α1-2Fuc |
| TJAII | *Trichosanthes japonica* | 43.6072 | 3.699 |  | 22.5096 | 4.147 | 0.000585 | α1-2Fuc |
| MCA | *Momordica charantia* | 40.2556 | 3.2644 |  | 50.4065 | 5.247 | 0.107359 | α1-2Fuc |
| FLAG-EW29CH |  | 61.1572 | 10.176 |  | 54.257 | 7.724 | 0.594861 | 6-sulfo-Gal |
| PTLI | *Psophocarpus tetragonolobu* | 1.13263 | 0.3469 |  | 0.00463 | 0.003 | 0.009977 | αGalNAc (A, Tn) |
| GSLIA4 | *Griffonia simplicifolia* | 35.1273 | 4.9235 |  | 3.95737 | 0.964 | 0.000114 | αGalNAc (A, Tn) |
| rGC2 | *Geodia cydonium* (recombinant) | 578.526 | 103.59 |  | 237.007 | 41.09 | 0.009829 | α1-2Fuc (H), αGalNAc (A), αGal (B) |
| GSLIB4 | *Griffonia simplicifolia* | 22.8793 | 6.3035 |  | 2.59449 | 1.122 | 0.010557 | αGal (B) |
| rMOA | *Marasmius oreades* (recombinant) | 96.0906 | 26.061 |  | 12.4358 | 2.703 | 0.010666 | αGal (B) |
| EEL | Euonymus europaeus | 37.9317 | 14.581 |  | 1.68356 | 0.989 | 0.034748 | αGal (B) |
| rPAIL | *Pseudomonas aeruginosa* (recombinant) | 21.2436 | 5.6702 |  | 2.16651 | 0.856 | 0.008314 | α,βGal, αGalNAc (Tn) |
| LEL | *Lycopersicon esculentum* | 58.1243 | 7.2893 |  | 49.2461 | 2.965 | 0.281021 | polylactosamine, (GlcNAc)n |
| STL | *Solanum tuberosum* | 166.929 | 21.289 |  | 113.323 | 7.586 | 0.036308 | polylactosamine, (GlcNAc)n |
| rGal3C | *Homo sapiens* (recombinant) | 297.222 | 15.029 |  | 186.892 | 13.46 | 1.05E-05 | LacNAc, polylactosamine |
| rLSL | *Laetiporus sulphureus* (recombinant) | 288.104 | 8.6053 |  | 245.202 | 8.139 | 0.001179 | LacNAc, polylactosamine |
| rCGL3 | *Coprinopsis cinerea* (recombinant) | 0.07068 | 0.0422 |  | 0.06459 | 0.022 | 0.900303 | LacdiNAc |
| PNA | *Arachis hypogaea* | 0.13096 | 0.0855 |  | 0.06597 | 0.023 | 0.479209 | Galβ1-3GalNAc (T) |
| ACA | *Amaranthus caudatus* | 104.428 | 13.755 |  | 150.149 | 7.782 | 0.010962 | Galβ1-3GalNAc (T) |
| HEA | *Hericium erinaceum* | 9.46889 | 2.8034 |  | 11.7111 | 1.208 | 0.476114 | Galβ1-3GalNAc (T) |
| ABA | *Agarics bisporus* | 40.7851 | 9.716 |  | 41.5416 | 4.558 | 0.944856 | Galβ1-3GalNAc (T), GlcNAc |
| Jacalin | *Artocarpus integrifolia* | 56.0417 | 9.6577 |  | 65.2225 | 4.808 | 0.409279 | Galβ1-3GalNAc (T), GalNAc (Tn) |
| MPA | *Maclura pomifera* | 20.0671 | 5.1621 |  | 7.29913 | 2.316 | 0.04208 | Galβ1-3GalNAc (T), GalNAc (Tn) |
| HPA | *Helix pomatia* | 150.4 | 60.191 |  | 86.0292 | 17.78 | 0.32786 | αGalNAc (A, Tn) |
| VVA | *Vicia villosa* | 0 | 0 |  | 0.00873 | 0.009 | 0.323475 | α,βGalNAc (A, Tn, LacdiNAc) |
| DBA | *Dolichos biflorus* | 19.7046 | 8.1741 |  | 2.03719 | 0.587 | 0.059166 | α,βGalNAc (A, Tn, LacdiNAc) |
| SBA | *Glycine max* | 4.04095 | 2.1486 |  | 0.35591 | 0.152 | 0.120946 | α,βGalNAc (A, Tn, LacdiNAc) |
| rPPL | *Pleurocybella porrigens* (recombinant) | 0.50844 | 0.3575 |  | 0.16224 | 0.058 | 0.362918 | α,βGalNAc (A, Tn, LacdiNAc) |
| rCNL | *Clitocybe nebularis* (recombinant) | 155.559 | 44.868 |  | 76.6504 | 13.2 | 0.12072 | α,βGalNAc (A, Tn, LacdiNAc) |
| rXCL | *Xerocomus chrysenteron* (recombinant) | 114.087 | 18.181 |  | 120.667 | 7.456 | 0.743439 | Core1,3, agalacto *N*-glycan |
| VVAⅠ | *Vicia villosa* | 0.00676 | 0.0068 |  | 0 | 0 | 0.343436 | GalNAcβ1-3(4)Gal |
| WFA | *Wisteria floribunda* | 6.501 | 2.302 |  | 3.66942 | 0.994 | 0.279885 | Terminal GalNAc, LacdiNAc |
| rABA | *Agarics bisporus* (recombinant) | 60.0584 | 13.903 |  | 59.5968 | 6.147 | 0.976243 | Galβ1-3GalNAc (T), GlcNAc |
| rDiscoidin I | *Dictyostelium discoideum* (recombinant) | 17.3072 | 3.3963 |  | 8.42636 | 1.492 | 0.032852 | Gal |
| DBAIII | Dioscorea batatas | 0 | 0 |  | 0 | 0 | - | Maltose |
| rMalectin | *Homo sapiens* (recombinant) | 0 | 0 |  | 0 | 0 | - | Glcα1-2Glc |
| CSA | *Oncorhynchus keta* | 0 | 0 |  | 0 | 0 | - | Rhamnose, Galα1-4Gal |
| FLAG-EW29Ch-E20K |  | 5.44968 | 2.7395 |  | 0.09056 | 0.066 | 0.082173 | 6-sulfo-Gal |
|  |  |  |  |  |  |  |  |  |
| *1*abbreviations: Fuc (L-fucose), Gal (D-galactose), GalNAc (N-acetyl-galactosamine), Glc (D-glucose), GlcNAc (N-acetyl-glucosamine), LacNAc (N-acetyl-lactosamine), Sia (sialic acid). *2*Specificity data was obtained by frontal affinity chromatography and glycoconjugate microarray. | | | | | | | | |
